# Chromone derivatives suppress neuroinflammation and improve mitochondrial function in the sporadic form of Alzheimer’s disease under experimental conditions

**DOI:** 10.22038/IJBMS.2022.65377.14387

**Published:** 2022-07

**Authors:** Dmitry I. Pozdnyakov, Denis S Zolotych, Viktoriya M Rukovitsyna, Eduard T Oganesyan

**Affiliations:** 1 Department of Pharmacology with Course of Clinical Pharmacology Pyatigorsk Medical Pharmaceutical Institute - A Branch of VolgGMU of the Ministry of Health of Russia, Pyatigorsk, Kalinin Ave., 11, 357532, Russia; 2 Department of Analytical Chemistry, Pyatigorsk Medical Pharmaceutical Institute - A Branch of VolgGMU of the Ministry of Health of Russia, Pyatigorsk, Kalinin Ave., 11, 357532, Russia; 3 Department of Organic Chemistry, Pyatigorsk Medical Pharmaceutical Institute - A Branch of VolgGMU of the Ministry of Health of Russia, Pyatigorsk, Kalinin Ave., 11, 357532, Russia

**Keywords:** Alzheimer’s disease, Chromone derivatives, Mitochondrial dysfunction, Neuroinflammation, Neuroprotection

## Abstract

**Objective(s):**

The study aims to estimate the neuroprotective effect of chromone derivatives in the sporadic form of Alzheimer’s disease in the context of the relationship between changes in mitochondrial function and neuroinflammation.

**Materials and Methods:**

Alzheimer’s disease was modeled by injecting Aβ 1-42 fragments into the CA1 part of the hippocampus of animals. The test compounds and memantine were administered orally for 60 days from the moment the pathology was reproduced. The change in cognitive deficit in rats was assessed in the Y-maze test. In the hippocampus of rats, intensity of cellular respiration, activity of mitochondrial enzymes (citrate synthase, aconitase, cytochrome-c-oxidase, and succinate dehydrogenase), concentrations of IL - 6; IL -1β; TNF -α; IL – 10, and cardiolipin were determined.

**Results:**

Of the 18 substances, only C3AACP6 and C3AACP7 compounds contributed to the recovery of aerobic metabolism, increased activity of mitochondrial enzymes, and reduced neuroinflammation in the hippocampus of rats. Furthermore, administration of these substances reduced the manifestation of cognitive deficit in animals with Alzheimer’s disease to a degree comparable with memantine. Moreover, in terms of the effect on changes in the activity of mitochondrial enzymes and aerobic metabolism, these substances significantly exceeded memantine.

**Conclusion:**

The study showed that from the analyzed chromone derivatives, two compounds C3AACP6 and C3AACP7 could have a neuroprotective effect in Alzheimer’s disease, which is realized through the axis: recovery of mitochondrial function, decrease extramitochondrial cardiolipin, decrease neuroinflammation, neuroprotection.

## Introduction

Alzheimer’s disease (AD) is one of the most common forms of terminal dementia, affecting more than 1.3 million people annually. To date, there are more than 55 million people in the world who suffer from AD, and the vast majority (over 60%) of them live in relatively economically prosperous regions, i.e., in high or middle-income countries. WHO and Alzheimer’s Disease International experts forecast an increase in the number of patients with AD to 139 million by 2050. It is quite indicative of the level of mortality from AD. Thus, in people older than 65%, AD is the fifth most common cause of death, and in recent years, the death rate from AD has increased by 146%. Whereas, for “millennium diseases”, such as arterial hypertension, coronary heart disease, or ischemic stroke, this indicator in the period 2009–2019 significantly decreased (1). Along with the progressive increase in morbidity and mortality, an important point in understanding what AD represents for the population is the increasing economic burden that affects both systemically (healthcare organizations) and persons providing direct care for patients with AD. Only in the United States, more than 16 billion working hours are annually spent by social services on helping patients with AD, which is equivalent to an economic cost of more than 270 billion dollars (2). Over the past few decades, some progress has been made in understanding the pathogenesis, diagnosis, and risk factors of AD. Most of the main points of the mechanisms of the pathological process development have been established, including amyloidogenesis, tauopathy, mitochondrial dysfunction, and genetic abnormalities. However, despite this, there are practically no medicines, the use of which in AD would reduce the intensity of the neurodegenerative process or restore clinically significant cognitive functions. As a rule, the treatment of AD comes down to strategies of non-drug and drug treatment. Non-pharmacological approaches to the treatment of AD include changes in lifestyle, social contacts and environment, stress reduction, and improved interaction in the care-patient system, for example, through psycho-education of medical service personnel caring for patients with AD. Drug treatment of AD is reduced to elimination of the symptoms of the disease, primarily cholinergic deficiency, which is achieved by the use of anticholinesterase and anti-excitotoxic drugs. To date, from the large collection of anticholinesterase drugs, rivastigmine (including in prolonged dosage forms), donepezil, and galantamine are the most widely used in the treatment of AD. Among the anti-excitotoxic agents, the blocker of NMDA receptors stands out. These groups of drugs must have a synergistically additive effect and complementary mechanisms of action, which makes it possible to achieve high rates of effectiveness in their combined use. The meta-analysis provided by Atri* et al., 2013,* showed that the combined use of donepezil and memantine in patients with mild and moderate AD alleviates the manifestations of cognitive deficit in the 24-week observation interval (3). In the monotherapy mode, these drugs also demonstrate an acceptable level of efficacy. Conducted randomized clinical trials show that acetylcholinesterase inhibitors improve the clinical status of patients with AD in the presence of a mild form of the disease (slow the progression of cognitive impairment), which in some cases reduces the burden on caregivers (4). Memantine demonstrates similar efficacy, as also evidenced by data from randomized clinical trials (5). However, it should be emphasized that there are practically no convincing results on the effectiveness of the long-term use of these drugs. It is also necessary to note the need for dose titration in almost every patient on an individual basis and the presence of quite serious adverse reactions (6). In this regard, the search for new therapeutic agents intended for the treatment of AD becomes relevant. According to Pleen & Townley, 2022, between 2019 and 2021, 226 interventional clinical trials and 51 observational studies were completed on the development of new treatments for AD (7). The discovery of the role of β-amyloid (Aβ) and the associated neurodestruction reactions in the pathogenesis of AD served to a large extent as the “research boom” of AD and methods of its treatment. A relationship has been established between increased content of Aβ and the development of tau-pathology, mitochondrial dysfunction, oxidative stress, neuroinflammation, and impaired synaptic transmission. However, the question of whether Aβ is a trigger of the neurodegenerative process or is secondary to other elements of the brain damage cascade in AD remains debatable, but despite this, the special role of Aβ in pathogenesis is beyond doubt (8). It is not surprising that new candidate molecules are currently being tested (9). The main vector for the development of anti-amyloid agents is the creation of monoclonal antibodies to Aβ. So, Bapineuzumab, Gantenerumab, and Crenezumab are currently undergoing clinical trials; however, the primary data obtained do not allow for discussing their undeniable therapeutic advantages (10).

In addition to monoclonal anti-Aβ antibodies, it is possible to reduce the formation of Aβ in brain tissue through the use of multipurpose small molecules, as indicated by Kumar* et al., *2022 (11). Chromone derivatives stand out among such molecules. Chromone derivatives are known for their neurotropic activity, including the presence of inhibitory activity against monoamine oxidase and acetylcholinesterase; they can also exhibit anti-excitotoxic properties (12). Previous studies have shown that the use of chromone derivatives under conditions of cerebral ischemia helps to reduce the degree of neuronal damage, which implies a pronounced neuroprotective effect (13). In this regard, we have synthesized new compounds - representatives of chromone derivatives, potentially having a pronounced neuroprotective effect.

The study aimed to investigate the neuroprotective effect of new chromone derivatives in conditions of Alzheimer’s disease caused by introduction of the Aβ _1-42 _fragment into the hippocampus of laboratory animals.

## Materials and Methods

***Animals***

Wistar rats (210 individuals) were obtained from the nursery of laboratory animals, Rappolovo. During the study, the animals were housed in the premises of the laboratory of living systems of the Pyatigorsk Medical and Pharmaceutical Institute under controlled environmental conditions: normal atmospheric pressure, air temperature 22–24 °С, air humidity 55–75%, and a 12-hr day/night cycle. The rats were placed in groups of 5 in polypropylene boxes, which were supplied with drinking accessories. The animals were kept on a complete diet with free access to water and feed. All animal procedures followed the generally accepted rules for the humane treatment of laboratory animals, as set out in Directive 2010/63/ EC of the European Parliament and the Council for the Protection of Animals used for Scientific Purposes, dated 22 September 2010 and ARRIVE 2.0 (14). The study design was reviewed and approved at a meeting of the local independent ethics committee of the Pyatigorsk Medical and Pharmaceutical Institute (Min Paper No. 10 dated February 19, 2021).

***Chemicals***

The test substances were obtained at the Department of Organic Chemistry, PMFI. The structures of the substances were confirmed by physicochemical methods of analysis and NMR spectroscopy. A complete description and identification of the studied substances are presented in Rukovitsina *et al*. (15). Reagents and solvents for the study were provided by Sigma - Aldrich (Germany) unless otherwise indicated. ELISA kits were provided by Cloud Clone (USA). The reference drug memantine was purchased from OZON Pharma (Russia).

***Study design***

During the study, the animals were divided into 21 equal groups of 10 animals each: sham-operated animals (SO), negative control (NC), a group of rats that were administered memantine at a dose of 30 mg/kg orally (16), and groups of animals (groups 4-21) treated with test compounds at a dose of 40 mg/kg (15), orally. The administration of the studied substances and the referent was carried out after modeling AD for 60 days. The administration was made through an atraumatic oral gavage once a day. After the specified time, the cognitive functions of rats in the Y-maze test were evaluated. Then the biomaterial was collected (the hippocampus), in whose tissue the change in the concentration of cytokines (IL-6; IL-1β; TNF-α; IL-10), the intensity of cellular respiration processes, and mitochondrial enzymes (citrate synthase, aconitase, cytochrome c-oxidase, and succinate dehydrogenase) were investigated. The content of cardiolipin was also determined in the hippocampus tissue. The study design is presented in Table 2.

***Experimental model of Alzheimer’s disease***

AD in Wistar rats was reproduced by direct injection of Aß _1-42 _aggregates into the hippocampus of the animals. Before injection, A ß _1-42 _fragments were solubilized in cold PBS pH=7.4 for 36 hr with constant stirring with an overhead mechanical stirrer until A ß _1-42 _aggregates were obtained. Next, the rats were anesthetized by intraperitoneal injection of chloral hydrate at a dose of 350 mg/kg, the parietal region was scalped, and the head of the rats fixed in a stereotaxis. Aggregates A ß _1-42 _were injected in a CA1 part of the hippocampus at a final concentration of 1 mmol/l in a volume of 2 µl using a microdoser and a G30 needle (anterior-posterior = -3.8 mm, medial-lateral = 2.0 mm, dorsal-ventral = 2.6 mm from the bregma, as determined by Paxinos and Watson 2007) (17). The needle was left in the injection site for 5 min, after which the tissue topography was restored and the wounds were sutured. The suture was treated with a 10% povidone-iodine solution (18).

***Assessment of cognitive functions in the test Y-shaped maze***

Sixty days after the operation, the rats were assessed for cognitive functions in the Y-maze test (19). The setup consisted of three equal arms connected at an angle of 120°. The animal was placed in the center of the device and the number of animal movements between the arms was recorded for 8 min. At the same time, spontaneous alternating entries into the arms (1-2-3, 3-1-2, 2-3-1) were recorded. Based on the obtained data, the percentage of spontaneous alternation was determined, which reflects the change in the cognitive abilities of animals:

Spontaneous alternation percentage *100

***Sampling biomaterial for analysis***

On the 61^st ^day of the study, the rats were decapitated (under anesthesia) and the brain was removed, which was placed in a cold-water bath at a temperature of 4 °C. The brain was dissected with a scalpel along the middle sulcus and the hippocampus was isolated. The animal hippocampus was homogenized in a mechanical homogenizer in a cold buffer solution consisting of 1 mM EGTA, 215 mM mannitol, 75 mM sucrose, 0.1% BSA solution, 20 mM HEPES, with a pH of 7.2 (tissue weight/buffer volume ratio was 1:7), to obtain a primary homogenate, which was divided into two parts. The first aliquot of the homogenate was centrifuged at 10,000 g for 15 min. The resulting supernatant was removed for an ELISA study. The second part of the homogenate was centrifuged at 1100 g for 2 min. The supernatant was transferred into Eppendorf tubes and layered with 10% Percoll solution, after which it was centrifuged again at 18,000 g for 10 min. The secondary supernatant was discarded, the pellet was resuspended in buffer solution, and re-centrifuged for 5 min, at 10,000 g. Changes in the processes of cellular respiration and the activity of mitochondrial enzymes were evaluated in the resulting supernatant (20).

***Study of cellular respiration processes***

Changes in the activity of cellular respiration processes were performed using an AKPM-1-01L laboratory respirometer to measure changes in oxygen consumption (OCR) with the addition of mitochondrial respiration uncouplers. The analyzed biomaterial was introduced into the respirometer cuvette, the substrate - pyruvic acid at a concentration of 15 mmol/ml was added and the basal level of OCR was fixed, after which uncouplers were added in turn: oligomycin 1 µg/ml; 4 - (trifluoromethoxy) phenyl) hydrazono) malononitrile (FCCP-1 µM/ml); rotenone - 1 µM/ml; sodium azide - 20 mM / ml. After each addition of an uncoupler to the analyzed mixture, the change in OCR was recorded. Based on received data ATP-generating ability (by the difference in OCR after the addition of FCCP and oligomycin), the maximum level of respiration (by the difference in OCR after the addition of FCCP, and rotenone), and the respiratory capacity (by the difference OCR after the addition of FCCP and the basal OCR level) were calculated (20).

***Evaluation of citrate synthase (CS) activity***

The citrate synthase activity was evaluated according to the method proposed by Shepherd & Garland*. *The method is based on the determination of colored products of 5,5’-di-thiobis- (2-nitrobenzoic acid) degradation. The analysis was carried out in a reaction medium consisting of: 5.5’- di-thiobis- (2-nitrobenzoic acid) 100 mM/l, acetyl CoA 100 mM/l; 0.1% Triton-X 100 µl /l, and 4 µl of the supernatant. The pH of the analyzed mixture was adjusted to 7.2 by adding Tris-HCl buffer solution. The reaction was started by adding 100 µl of oxaloacetate. The change of absorbance was recorded at a wavelength of 412 nm for 3 min at room temperature. Citrate synthase activity was expressed in U/mg protein. Protein concentration was determined by the Bradford method (21).

***Determination of aconitase (Aco) activity***

Aconitase activity was assessed by the degree of NADPH formation in the conjugated reaction, which is catalyzed by aconitase and isocitrate dehydrogenase. The analyzed media settled of the tested biomaterial-50 µl (or aconitase 0.03 U/L in a positive control sample), isocitrate dehydrogenase 0.03 U/L, NADP ^+ ^0.32 mg/ml. The sample was adjusted to 100 µl with phosphate buffer solution. The reaction was started by adding 0.1 mg/ml of sodium citrate. Changes in the optical density of the obtained solutions were registered at 340 nm, 37 °C for 2 min. Aconitase activity was calculated from changes in optical density using the extinction coefficient 0.0313 µM ^-1 ^(22).

***Succinate dehydrogenase (SDH) activity evaluation***

The activity of succinate dehydrogenase was assessed spectrophotometrically in the reaction of succinate-dependent reduction of dichlorophenolindophenol when rotenone was added to the analyzed medium at 600 nm. During the analysis, standard kits from Abcam were used. Absorbance was recorded on an Infinite F50 reader (Tecan, Austria).

***Cytochrome c oxidase (COX) activity evaluation***

The activity of cytochrome c oxidase was determined in the mitochondrial fraction by the change in the optical density of the medium of the oxidation reaction of cytochrome C (II) in the presence of KCN at 500 nm. Standard kits from Abcam were used in the analysis. Absorbance was recorded on an Infinite F50 reader (Tecan, Austria).

***ELISA study***

In the study, the concentrations of Aβ, cytokines (IL-6; IL- 1β; TNF- α; IL-10), and cardiolipin were determined by ELISA in the supernatant of the hippocampus. The preparation of samples and the analysis progress corresponded to the instructions attached to each kit.

***Statistical analysis***

The results were statistically processed using the STATISTICA 6.0 software package (StatSoft). Data were expressed as M ± SEM (mean ± standard error of the mean). The normality was assessed using the Shapiro-Wilk test, and the uniformity of variance was assessed using Leven’s test. The statistical significance of the differences between the groups was carried out by the method of one-way analysis of variance with post-processing by the Newman-Keuls test at a critical level of significance *P*<0.05.

## Results

***The effect of the tested compounds on the change in the cognitive deficit of animals under experimental AD***

**Figure 1:**
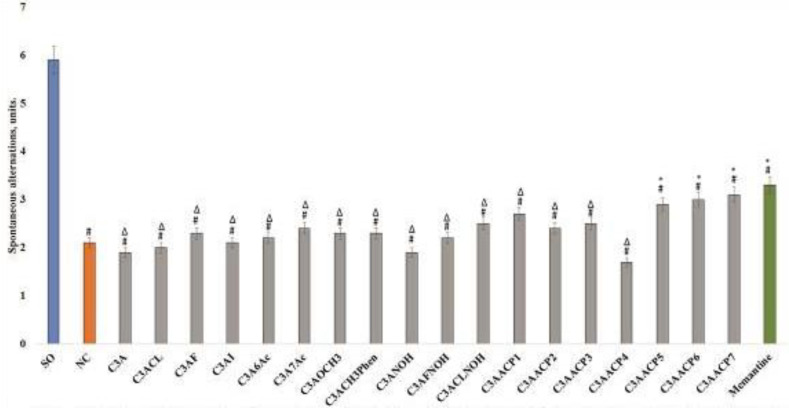
Influence of test compounds and memantine on the change of behavior of animals in the Y-maze test under conditions of experimental Alzheimer’s disease (AD)

Note: # - significant relative to the sham-operated (SO) group (Newman-Keuls test, *P*<0.05); * - significant relative to the negative control (NC) group (Newman-Keuls test, *P*<0.05); ?- significant relative to the memantine rats (Newman-Keuls test, *P*<0.05)

It was found that in rats of the NC group (Figure 1) when Aβ _1-42 _aggregates were injected into the hippocampus, the development of pronounced cognitive impairment was noted. In this group of animals, when tested in a Y-shaped maze, a decrease in spontaneous exploratory activity by 64.4% (*P*<0.05) relative to the SO of rats was noted. The course administration of memantine to animals prevented the formation of cognitive deficit and, as a result, the percentage of spontaneous alternations of the labyrinth arms in animals treated with memantine was 57.1% higher than in NC rats (*P*<0.05). The analyzed chromone derivatives under the codes C3AACP5, C3AACP6, and C3AACP7 demonstrated comparable activity with the reference drug. So, against the administration of these substances to rats with AD, the severity of the violation of the integrative functions of the brain decreased in comparison with the NC group of animals by 38.0%, 42.9%, and 47.6% (all *P*<0.05), which were not significantly different from the activity scores of animals treated with memantine. The use of the rest of the studied compounds did not have a significant effect on the change in the cognitive functions of the animals, and the results of spontaneous exploratory activity in these groups did not significantly differ from the NC group of rats and were inferior to the animals that received the reference drug (Figure 1).

***The effect of the tested compounds on the change of the Aβ concentration in the hippocampus of animals under conditions of experimental AD***

**Figure 2:**
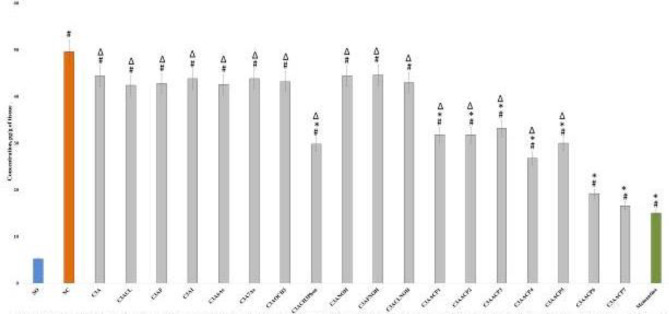
Influence of test compounds and memantine on the change of amyloid beta in the hippocampus of animals under conditions of experimental Alzheimer’s disease (AD)

Note: # - significant relative to the sham-operated (SO) group (Newman-Keuls test, *P*<0.05); * - significant relative to the negative control (NC) group (Newman-Keuls test, *P*<0.05); ? - significant relative to the memantine rats (Newman-Keuls test, *P*<0.05)

Since the accumulation of Aβ aggregates is a hallmark of the pathogenesis of AD, we studied the effect of chromone derivatives on changes in the concentration of Aβ in the hippocampus in rats (Figure 2). As a result, it was shown that in the NC group of rats, the content of Aβ in the hippocampus was 9.5 times higher than in the rats of the SO group by 9.5 times (*P*<0.05). The ongoing therapy by the studied chromone derivatives and memantine demonstrated different levels of effectiveness. So, against the background of the course administration of the reference drug to animals, a decrease in the concentration of Aβ in the hippocampus by 69.8% was observed, and the changes were significant. When using chromone derivatives under the codes C3AACP1 - C3AACP7, the content of Aβ, as well as in the case of administration of memantine, was statistically significantly less than that in NC animals, while significant differences between the groups of rats treated with compounds C3AACP6 and C3AACP7 and memantine were not established. It should be noted that administration of the C3ACH3Phen substance led to a decrease in the Aβ content in the hippocampus of rats by 38.9% (*P*<0.05), while the use of other chromone derivatives showed a low degree of therapeutic efficacy, significantly inferior to memantine (Figure 2).

***Influence of the tested compounds on the change in the intensity of cellular respiration in the hippocampus of animals under conditions of experimental AD***

Studies of recent decades have established that alteration of aerobic metabolism plays a significant role in the progression of neurodegenerative diseases, including Alzheimer’s pathology. An integral assessment of the change in the state of energy production in cells is possible by determining the change in OCR when the cell mitochondria switch to various energy-synthesizing states, which is achieved by introducing uncouplers of mitochondrial respiration into the analyzed system (23). Conducted cellular respirometry showed significant deterioration in energy production in the hippocampal tissue in the NC group of rats, in which ATP-generating activity, the maximum level of respiration, and respiratory capacity were lower than those in SO animals by 45.7%, 52.4%, and 53.1%, respectively (all *P*<0.05). Among the analyzed substances, the most significant effect on the change in cellular respiration was exerted by the use of substances under the codes C3AACP4-C3AACP7, against the background of which the ATP-generating activity, the maximum level of respiration, and respiratory capacity were statistically significantly higher than those in the NC group of animals. A comparable level of activity was shown by the reference drug memantine, the use of which led to an increase in ATP-generating activity by 33.8%, the maximum level of respiration by 24.9% and respiratory capacity by 52.9% (all indicators significantly differed from the NC group animals). The intensity of cellular respiration against the background of the introduction of other studied substances was similar to the NC group of rats without statistically significant differences (Table 3).

***Influence of the tested compounds on changes in the activity of mitochondrial enzymes in the hippocampal tissue of animals under conditions of experimental AD***

Cellular respiration and the formation of macroergic compounds are inextricably linked with a change in the functional activity of mitochondria and, in particular, mitochondrial enzyme systems responsible for the optimal flow of ATP biosynthesis, inactivation of reactive oxygen species, and mitochondrial biogenesis.

In rats with experimental AD without treatment, a statistically significant decrease in the activity of mitochondrial enzymes was observed (Table 4) in relation to SO rats, while the activity of citrate synthase decreased by 65.2% ( *P*<0.05), aconitase by 38.4% ( *P*<0.05), succinate dehydrogenase by 51.2% (*P*<0.05), and cytochrome c-oxidase by 43.8% (*P*<0.05). The course administration of memantine to animals contributed to an increase in the activity of citrate synthase by 26.4% (*P*<0.05), while the activity of the other evaluated enzymes did not statistically significantly differ from that of the NC group of animals (Table 4). On the contrary, the use of the studied compounds had a more pronounced effect on changes in the activity of mitochondrial enzymes. In particular, in rats treated with compounds C3ACH3Phen and C3AACP1-C3AACP 7, the activity of citrate synthase, aconitase, succinate dehydrogenase, and cytochrome- c-oxidase was significantly (*P*<0.05) higher than that of both the NC group of animals and the group of rats that received the reference drug. The use of other chromone derivatives also led to an increase in the activity of mitochondrial enzymes, with the exception of cytochrome-c-oxidase, but in terms of activity, they were inferior to the compounds C3ACH3Phen and C3AACP1-C3AACP7.

***Influence of the tested compounds on changes in the processes of neuroinflammation and cardiolipin concentration in the hippocampal tissue of animals under conditions of experimental AD***

Neuroinflammation, as well as the amyloid cascade, is a significant component of AD pathogenesis. In this regard, we studied the effect of chromone derivatives on changes in the content of pro/anti-inflammatory cytokines in the rat hippocampal tissue. The concentration of cardiolipin was also determined as one of the factors activating neuroinflammation (24).

As a result, it was found that in the NC group of animals, when modeling AD, the content of pro-inflammatory cytokines (Table 5) in the hippocampal tissue was statistically significantly higher than that in SO rats. In the NC animal group, the concentrations of IL-6, IL-1β, and TNF-α increased by 100.8% (*P*<0.05), 74.3% (*P*<0.05), and 209.6% (*P*<0.05), while the content of IL-10 decreased by 28.5% (*P*<0.05). Administration of memantine resulted in a significant decrease in the content of IL-6 and IL-1β by 19.8% and 33.0% compared with the NC group of animals. Among the studied chromone derivatives, neuroinflammation reactions were most significantly suppressed when using compounds under the codes C3ACH3Phen, C3AACP4, C3AACP5, C3AACP6, and C3AACP7, against the background of the administration of which the content of IL- 6, IL-1β and TNF-α decrease. It should be noted that neither the use of the reference drug nor the introduction of the analyzed substances had a significant effect on the change in the concentration of IL-10.

**Figure 3:**
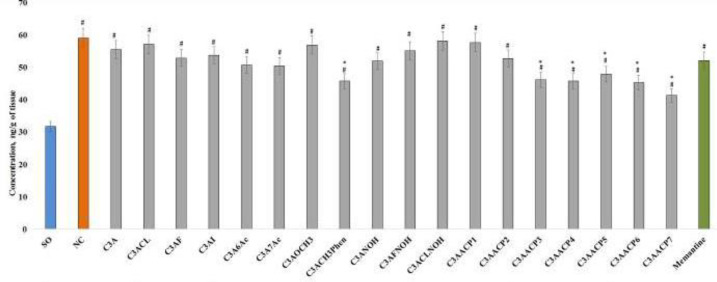
**I**nfluence of test compounds and memantine on the change of cardiolipin concentration in the hippocampus of animals under conditions of experimental lzheimer’s disease (AD)

Note: # - significant relative to the sham-operated (SO) group (Newman-Keuls test, *P*<0.05); * - significant relative to the negative control (NC) group (Newman-Keuls test, *P*<0.05)

In the course of assessing changes in the content of cardiolipin in the hippocampal tissue in rats with AD, a significant increase in its concentration in the NC group of animals by 86.2% relative to the SO rats was found (Figure 3). Among the studied compounds, only the use of substances under the codes C3ACH3Phen and C3AACP3 - C3AACP7 contributed to a decrease in the content of cardiolipin by 22.8%; 22.0%; 22.6%; 18.9%; 23.3%; and 30.1%, respectively (all *P*<0.05). It should be noted that administration of memantine and other test substances to animals was not accompanied by a significant change in the concentration of cardiolipin (Figure 3).

**Table 1:** Test compounds (chromone derivatives) for investigation

**IUPAC chemical name**	**Laboratory code**
3-formylchromone	C3A
6-chloro-4-oxo-4H-1-benzopyran-3-carbaldehyde	C3ACL
6-fluoro-4-oxo-4H-1-benzopyran-3-carbaldehyde	C3AF
6-iodo-4-oxo-4H-1-benzopyran-3-carbaldehyde	C3AI
3-formyl-4-oxo-4H-1-benzopyran-6-yl acetate	C3A6Ac
3-formyl-4-oxo-4H-1-benzopyran-7-yl acetate	C3A7Ac
3-methoxy-4-oxo-4H-1-benzopyran-3-carbaldehyde	C3AOCH3
2-[methyl(phenyl)amino]-4-oxo-4H-1-benzopyran-3-carbaldehyde	C3ACH3Phen
3-[(E)-(hydroxyimino)methyl]-4H-1-benzopyran-4-one	C3ANOH
(3E) -6-fluoro-4-oxo-chromene-3-carbaldehyde oxime	C3AFNOH
(3E) -6-chloro-4-oxo-chromene-3-carbaldehyde oxime	C3ACLNOH
3-[(1E)-3-oxo-3-phenylprop-1-en-1-yl]-4H-1-benzopyran-4-one	C3AACP1
3-[(1E)-3-(3,4-dimethylphenyl)-3-oxoprop-1-en-1-yl]-4H-1-benzopyran-4-one	C3AACP2
3-[(1E)-3-(2-hydroxy-5-methylphenyl)-3-oxoprop-1-en-1-yl]-4H-1-benzopyran-4-one	C3AACP3
3-[(1E)-3-(5-fluoro-2-hydroxyphenyl)-3-oxoprop-1-en-1-yl]-4H-1-benzopyran-4-one	C3AACP4
3-[(1E)-3-(2-hydroxy-3-iodo-5-methylphenyl)-3-oxoprop-1-en-1-yl]-4H-1-benzopyran-4-one	C3AACP5
3-[(1E)-3-(2-hydroxy-4-methoxyphenyl)-3-oxoprop-1-en-1-yl]-4H-1-benzopyran-4-on	C3AACP6
3-[(E)-3-(3,5-di-tert-butyl-4-hydroxy-phenyl)-3-oxo-prop-1-enyl]-6-methoxy-chromen-4-one	C3AACP7

**Table 2:** Study design for chomone derivatives

**Group**	**Procedure**		
SO (n=10)	-	-	Sampling of biomaterial, sample preparation, and determination of the studied parameters.
NC (n=10)	AD modeling	Administration of purified water (equivalent volume)	
Memantine (n=10) , 30 mg/kg		Administration of the tested substances and the referent for 60 days. Orally, daily, one administration per day.	
C3A (n=10), 40 mg/kg			
C3ACL (n=10), 40 mg/kg			
C3AF (n=10), 40 mg/kg			
C3AI (n=10), 40 mg/kg			
C3A6Ac (n=10), 40 mg/kg			
C3A7Ac (n=10) 40 mg /kg			
C3AOCH3 (n=10), 40 mg/kg			
C3ACH3Phen (n=10), 40mg /kg			
C3ANOH (n=10), 40 mg/kg			
C3AFNOH (n=10), 40 mg/kg			
C3ACLNOH (n=10), 40 mg/kg			
C3AACP1 (n=10), 40 mg/kg			
C3AACP2 (n=10), 40 mg/kg			
C3AACP3 (n=10), 40 mg/kg			
C3AACP4 (n=10), 40 mg/kg			
C3AACP5 (n=10), 40 mg/kg			
C3AACP6 (n=10), 40 mg/kg			
C3AACP7 (n=10), 40 mg/kg			

SO - sham-operated animals; NC - negative control

**Table 3:** Influence of the tested compounds and memantine on the change in the intensity of cellular respiration in the hippocampal tissue of animals under conditions of experimental Alzheimer’s disease (AD)

**Group**	**ATP-generating ability, ppm/ mg protein**	**Maximal respiratory rate, ppm/ mg protein**	**Respiratory capacity, ppm/ mg protein**
SO	37.6±5.092	41.4±5.428	39.9±6.263
NC	20.4±4.157 #	19.7±5.238 #	18.7±6.928 #
C3A	21.0±4.195 #	27±5.109 #	21.7±4.86 #
C3ACL	20.6±5.557 #	23.3±6.12 #	21.7±5.088 #
C3AF	21.3±6.591 #	21.1±6.19 #	18.2±6.575 #
C3AI	22.6±3.384 #	20.9±5.471 #	21.6±4.045 #
C3A6Ac	22.2±6.015 #	22.3±6.392 #	22.1±4.24 #
C3A7Ac	21.6±5.216 #	22.5±4.936 #	20.9±6.669#
C3AOCH3	24.6±4.749#	20.5±5.178#	21.4±3.176#
C3ACH3Phen	19.5±6.252#	20.1±3.939#	20.2±6.236#
C3ANOH	20.5±3.581#	2 1.5±4.386 #	21.5±4.275 #
C3AFNOH	20.9±4.674 #	21.8±6.508 #	20.2±5.417 #
C3ACLNOH	22.6±3.642 #	21.8±6.201 #	21.8±6.639 #
C3AACP1	21.6±3.074 #	20.8±6.115 #	21.9±5.288 #
C3AACP2	22.4±3.624 #	24.3±6.973 #*	22.1 ± 6.931 #
C3AACP3	21.8±6.831 #	20.4±3.576 #*	19.8±4.786 #*
C3AACP4	25.2±4.045 #*	28.1±5.4 14#*	22.9±6.215 #*
C3AACP5	26.7±3.452 #*	22.9±5.738 #*	24.4±4.744 #*
C3AACP6	26.5±6.933 #*	24.7±5.861 #*	26.8±3.697 #*
C3AACP7	27.6±5.962 #*	26.9±4.746 #*	27.3±5.281 #*
Memantine	27.3±4.687 #*	24.9±3.926 #*	2 8 .6±2.391 #*

Note: # - significant relative to the sham-operated (SO) group (Newman-Keuls test, *P*<0.05); * - significant relative to the negative control (NC) group (Newman-Keuls test, *P*<0.05)

**Table 4:** Effect of the tested compounds and memantine on changes in the activity of mitochondrial enzymes in the hippocampal tissue of animals under conditions of experimental Alzheimer’s disease (AD)

**Group**	**CS , U/mg protein**	**Aco, U/mg protein**	**SDH, U/mg protein**	**COX, U/mg protein**
SO	3.71±0.885	17.22±0.592	2.93±0.638	3.15±0.387
NC	1.29±0.015 #	10.61±0.552 #	1.43±0.026 #	1.77±0.058 #
C3A	2.11±0.693 #*	11.62±0.822 #*	1.8±0.071 #*	1.79±0.094 #
C3ACL	2.05±0.056 #*	10.23±0.43 #*	1.7±0.064 #*	1.9±0.062 #
C3AF	0.13±0.062 #*	12.39±0.615 #*	1.59±0.084 #*	1.79±0.013 #
C3AI	2.08±0.169 #*	14.84±0.502 #*	1.74±0.038 #*	1.85±0.074 #
C3A6Ac	1.95±0.09 #*	14.71±0.979 #*	1.8±0.061 #*	1.74±0.039 #
C3A7Ac	1.85±0.075 #*	12.23±0.402 #*	2.2±0.02 #*	1.98±0.086 #
C3AOCH3	1.62±0.062 #*	13.55±0.224 #*	1.8±0.077 #*	2.01±0.053 #
C3ACH3Phen	1.73±0.053 #*	13.04±0.518 #*	1.69±0.071 #*	2.14±0.087 #
C3ANOH	1.59±0.071 #*	13.07±0.825 #*	1.75±0.051 #*	2.06±0.061 #
C3AFNOH	1.96±0.049 #*	13.12±0.924 #*	1.61±0.044 #*	1.56±0.091 #
C3ACLNOH	1.71±0.052 #*	12.74±0.436 #*	1.69±0.032 #*	1.79±0.041 #
C3AACP1	2.21±0.016 #* Δ	14.96±0.077 #* Δ	1.72±0.036 #* Δ	2.03±0.018 #* ∆
C3AACP2	2.26±0.166 #* Δ	14.64±0.7 #* ∆	1.86±0.099 #* ∆	2.05±0.052 #* ∆
C3AACP3	2.14±0.076 #* Δ	13.52±0.027 #* Δ	1.97±0.067 #* ∆	2±0.095 #* Δ
C3AACP4	2.21±0.094 #* Δ	13.27±0.045 #* Δ	2.01±0.072 #* ∆	1.95±0.036 #* Δ
C3AACP5	2.14±0.089 #* Δ	12.76±0.082 #* Δ	2.09±0.078 #* ∆	2.13±0.081 #* Δ
C3AACP6	2.23±0.012 #* Δ	14.29±0.041 #* Δ	2.1±0.053 #* ∆	2.12±0.045 #* Δ
C3AACP7	2.45±0.041 #* Δ	15.5±0.047 #* Δ	2.16±0.013 #* Δ	2.26±0.061 #* Δ
Memantine	1.63±0.548 #	11.97±0.022 #	1.67±0.706 #	1.86±0.839 #

Note: CS: citrate synthase; Aco: aconitase; SDH: succinate dehydrogenase; COX: cytochrome c oxidase; #: significant relative to the sham-operated (SO) group (Newman-Keuls test, *P*<0.05); * - significant relative to the negative control (NC) group (Newman-Keuls test, *P*<0.05).

**Table 5:** Influence of the tested compounds and memantine on changes in the concentration of cytokines in the hippocampal tissue of animals under conditions of experimental Alzheimer’s disease (AD)

**Group**	**IL-6 ng/ml**	**IL-1β, ng/ml**	**TNF-α, ng/ml**	**IL-10 ng/ml**
SO	0.126±0.043	0.167±0.018	0.135±0.065	0.295±0.032
NC	0.253±0.059 #	0.291±0.061 #	0.418±0.037 #	0.211±0.059 #
C3A	0.2 3 5±0.044 #	0.261±0.077 #	0.397±0.06 #	0.236±0.024 #
C3ACL	0.226±0.051 #	0.2791±0.026 #	0.374±0.078 #	0.245±0.027 #
C3AF	0.254±0.076 #	0.263±0.052 #	0.384±0.061 #	0.235±0.031 #
C3AI	0.221±0.014 #	0.274±0.038 #	0.365±0.034 #	0.248±0.018 #
C3A6Ac	0.222±0.036 #	0.297±0.049 #	0.397±0.073 #	0.246±0.012 #
C3A7Ac	0.213±0.032 #	0.258±0.068 #	0.361±0.011 #	0.243±0.024 #
C3AOCH3	0.232±0.02 #	0.2556±0.029 #	0.3 6 4±0.054 #	0.231±0.038 #
C3ACH3Phen	0.185±0.044 #*	0.207±0.012 #*	0.399±0.018 #*	0.235±0.053 #
C3ANOH	0.227±0.017 #	0.257±0.031 #	0.395±0.063 #	0.239±0.055 #
C3AFNOH	0.238±0.03 #	0.267±0.069 #	0.39 2 ±0.019 #	0.24±0.017 #
C3ACLNOH	0.22 7 ±0.043 #	0.274±0.069 #	0.369±0.041 #	0.236±0.019 #
C3AACP1	0.221±0.068 #	0.269±0.047 #	0.386±0.061 #	0.239±0.077 #
C3AACP2	0.229±0.07 #	0.186±0.02 #	0.374±0.069 #	0.245±0.061 #
C3AACP3	0.219±0.066 #	0.222±0.049 #	0.397±0.042 #	0.245±0.013 #
C3AACP4	0.195±0.056 #*	0.225±0.058 #*	0.31±0.041 #*	0.246±0.042 #
C3AACP5	0.193±0.058 #*	0.226±0.036 #*	0.325±0.072 #*	0.237±0.06 #
C3AACP6	0.197±0.028 #*	0.201±0.074 #*	0.365±0.07 #*	0.231±0.013 #
C3AACP7	0.183±0.057 #*	0.184±0.017 #*	0.326±0.038 #*	0.248±0.019 #
Memantine	0.203±0.026 #*	0.195±0.017 #*	0.351±0.089 #	0.241±0.065 #

Note: # - significant relative to the sham-operated (SO) group (Newman-Keuls test, *P*<0.05); * - significant relative to the negative control (NC) group (Newman-Keuls test, *P*<0.05)

## Discussion

AD is a terminal form of dementia, the spread of which has become rampant. Data provided by WHO experts show that AD is one of the leading causes of death in the population, second only to cardiovascular diseases (ischemic heart disease and ischemic stroke) in some cases. The treatment of AD is a complex therapeutic task that requires an integrated approach. Currently, AD therapy is reduced to the elimination of cholinergic deficiency through the use of anticholinesterase and antiglutamate agents (25). Thus, currently available strategies for the treatment of AD are focused on eliminating the symptoms of the disease and poorly affect the pathogenetic mechanisms of the development of AD. In many respects, this may be due to the complex stepwise cascade of the pathogenesis of the disease. It has been established that a distinctive feature of the mechanism of development of Alzheimer’s pathology is the deposition of Aβ aggregates in the brain structures, which can initiate secondary cascades of brain damage. For example, Aβ conglomerates can be recognized by the immune system as a foreign agent, as a result, microglial cells are activated, which produce pro-inflammatory cytokines, leading to cell death. This cascade is known as neuroinflammation and is one of the central aspects of AD pathogenesis (26). Neuroinflammation is primarily associated with the activation of microglial cells. Being extremely labile microglia cells quickly switch between two phenotypes: physiological M2 and pathological M1. Cells of the M1 phenotype form with Aβ inflammasome aggregates, which leads to an increase in the synthesis of cytokines such as IL-6, IL-1β, and TNF-α (27). The action of cytokines is aimed at elimination of damaged neurons, which provides a phase of compensation, and at a sufficiently low content of Aβ in brain structures, microglial cells pass to the M2 phenotype, limiting the neuroinflammatory process. However, in the presence of a pathological process of high intensity, microglial cells do not return to the anti-inflammatory phenotype, while neuroinflammation increases, leading to neurodegeneration (28). As a rule, the transition of glial cells to the M2 phenotype is prevented by the cytotoxic effects of cytokines, which through TRAIL receptors trigger neuronal apoptosis with the release of DAMPS (molecular patterns associated with damage), thereby closing the vicious circle of neuroinflammation (29). DAMPS are represented by an extensive pool of molecules, but the most significant contribution to the progression of neuroinflammation is made by the products of mitochondrial destruction, i.e., mitochondrial DAMPS (30).

Mitochondria are primarily known as the powerhouses of the cell, providing the synthesis of ATP in the process of oxidative phosphorylation. It is known that cytokines, primarily TNF-α, suppress the reactions of electron transfer along the mitochondrial respiratory chain, increase the production of ROS by mitochondria, and lead to the formation of giant pores on the inner mitochondrial membrane and its permeabilization. As a result, in addition to apoptosis activated by cytokines, an intracellular pathway dependent on mitochondria joins (31). Apoptotic cell death leads to the release of new DAMPS, which aggravates the inflammatory process in the nervous tissue. These molecules are represented by fragments formed during the destruction of mitochondria, for example, mtDNA or cardiolipin (32).

Thus, in the pathogenesis of AD, a clear relationship between amyloidogenesis, neuroinflammation, and mitochondrial dysfunction is assumed, which opens up certain prospects for the development of new pathogenetic strategies for the treatment of AD (33). To date, only sodium oligomannate (GV -971), the only representative of low-molecular-weight molecules that inhibit the formation of Aβ, is at the stage of clinical trials (34). At the same time, preclinical studies of new promising substances for the pathogenetic therapy of AD are being actively conducted. Literature data show that chromone derivatives can be such compounds. Thus, the present study was focused on evaluating the ability of chromone derivatives to exert a therapeutic effect in AD in the context of pathogenetic therapy. Eighteen compounds were analyzed in the model of the sporadic form of AD caused by the injection of Aβ _1-42 _fragments into the hippocampus of animals. As a result, the study showed that, in terms of the totality of the observed changes, only 2 compounds under the codes C3AACP6 and C3AACP7 in a number of studied substances have a pronounced therapeutic effect. The course administration of these substances contributed to the normalization of aerobic metabolism with an increase in the activity of mitochondrial enzymes succinate dehydrogenase, aconitase, citrate synthase, and cytochrome c-oxidase. An important aspect of the action of chromone derivatives, established in this study, is a decrease in the concentration of cardiolipin in the hippocampal tissue. It is known that cardiolipin is an important mitochondrial phospholipid involved in the organization of mitochondrial membranes, the formation of supercomplexes, the assembly of F1–F0 ATP synthase, and acts as a signal molecule during mitochondrial disorganization (35). As mentioned above, cardiolipin belongs to DAMPS, which activate neuroinflammation, an increase in the extracellular concentration of which indicates an intensification of cell damage processes (36). In this regard, the decrease in the content of cardiolipin observed against the background of the administration of the studied substances may indicate the preservation of the architectonics of mitochondria, which, at the level of the literature and obtained data, is consistent with an increase in the respirometric function of mitochondria and the activity of enzymes of mitochondrial origin (37). Also, when using the studied substances, especially C3AACP6 and C3AACP7, there was a decrease in the concentration of pro-inflammatory cytokines in the brain tissue, which may be a consequence of a decrease in the content of cardiolipin. The role of cardiolipin in the modulation of cytokine production by microglial cells is described by Wenzel* et al., 2021*. This authors’ team demonstrated that cardiolipin, through toll-like receptor 4 (TLR 4) increases the production of cytokines by microglial cells under pathological conditions and reduces their production under physiological conditions (38). Proinflammatory cytokines play a significant role not only in the progression of neuroinflammation but also in the formation of Aβ and its aggregates. Alastair* et al., 2018, *showed the ability of cytokines to activate enzymes of the secretase family (β and γ-secretases), which catalyze the cleavage of the amyloid precursor protein to pathologically significant Aβ particles, initiating amyloidogenesis (39). In this regard, a decrease in the content of cytokines in the brain tissue may underlie the decrease in the concentration of Aβ, which was also noted in this study.

An important point in the development of new drugs for the treatment of AD and, in general, neuroprotective agents is the improvement of the clinical status in the presence of a neurodegenerative process. In the context of AD, the improvement of the clinical symptoms of the disease comes down, first of all, to an increase in cognitive functions. In this study, it was found that the use of chromone derivatives eliminated cognitive deficits in rats in the Y-shaped maze test.

## Conclusion

The search for pathogenetic agents for the treatment of AD is an urgent task of modern medicine and pharmacology. Chromone derivatives may be promising multi-target agents for the treatment of AD, the evaluation of the effectiveness of which was the subject of this study. The results of this work indicate that the use of chromone derivatives is not inferior in terms of pharmacological effect to the reference drug memantine, and in some cases (for example, in terms of the effect on changes in the activity of mitochondrial enzymes) surpasses it. At the same time, the action of the analyzed chromone derivatives is described by the axis: restoration of the structural and functional activity of mitochondria → stabilization of mitochondrial membranes → decrease in cardiolipin releasing → decrease in cytokine production by glial cells and neuroinflammation → decrease in the Aβ content in the hippocampus → improvement in cognitive functions.

## Authors’ Contributions

EO and PD Developed the research concept; VR and DZ Synthesized the studied substances; PD Conducted the experiment; PD and DZ Processed the obtained data; PD and VR Prepared the final version of the manuscript; EO Critically reviewed the manuscript.

## Final Support

The study was performed without sponsorship.

## Conflicts of Interest

The authors declare that there are no conflicts of interest.
